# Correlation between Agar Plate Screening and Solid-State Fermentation for the Prediction of Cellulase Production by *Trichoderma* Strains

**DOI:** 10.1155/2012/793708

**Published:** 2012-11-28

**Authors:** Camila Florencio, Sonia Couri, Cristiane Sanchez Farinas

**Affiliations:** ^1^Embrapa Instrumentação, Laboratório de Agroenergia, Caixa Postal 741, 13560-970 São Carlos, SP, Brazil; ^2^Programa de Pós-graduação em Biotecnologia, Universidade Federal de São Carlos, 13565-905 São Carlos, SP, Brazil; ^3^Programa de Pós-graduação em Ciência e Tecnologia de Alimentos, Instituto Federal de Educação, Ciência e Tecnologia do Rio de Janeiro, Rua Senador Furtado 121, Maracanã, 20270-021 Rio de Janeiro, RJ, Brazil

## Abstract

The viability of converting biomass into biofuels and chemicals still requires further development towards the reduction of the enzyme production costs. Thus, there is a growing demand for the development of efficient procedures for selection of cellulase-producing microorganisms. This work correlates qualitative screening using agar plate assays with quantitative measurements of cellulase production during cultivation under solid-state fermentation (SSF). The initial screening step consisted of observation of the growth of 78 preselected strains of the genus *Trichoderma* on plates, using microcrystalline cellulose as carbon source. The 49 strains that were able to grow on this substrate were then subjected to a second screening step using the Congo red test. From this test it was possible to select 10 strains that presented the highest enzymatic indices (EI), with values ranging from 1.51 to 1.90. SSF cultivations using sugarcane bagasse and wheat bran as substrates were performed using selected strains. The CG 104NH strain presented the highest EGase activity (25.93 UI*·*g^−1^). The EI results obtained in the screening procedure using plates were compared with cellulase production under SSF. A correlation coefficient (*R* ^2^) of 0.977 was obtained between the Congo red test and SSF, demonstrating that the two methodologies were in good agreement.

## 1. Introduction

Cellulolytic microorganisms play an important role in the biosphere by recycling cellulose, the most abundant renewable carbohydrate produced by plants through the mechanism of photosynthesis [1]. In order to perform this task, these microorganisms have evolved a variety of strategies to attack the cell wall components, and therefore possess a comprehensive enzymatic arsenal that is able to degrade plant biomass. These enzymatic cocktails are often optimized according to the substrate and contain a mixture of cellulases, hemicellulases, pectinases, ligninases, and other accessory enzymes that act synchronously and synergistically in the degradation process [2]. Given the advantages of the enzymatic route in the bioconversion of biomass into fuels, there is an increasing demand for more effective enzymatic cocktails that could help to reduce the costs of cellulosic ethanol production.

Among a large number of nonpathogenic microorganisms capable of producing useful enzymes, filamentous fungi are particularly interesting due to their high production of extracellular enzymes [3]. Members of the *Trichoderma* genus are especially notable for their high enzymatic productivity. Around 100 different *Trichoderma* species have been identified. *Trichoderma reesei* is a major industrial source of cellulases and hemicellulases due to its ability to secrete large quantities of hydrolytic enzymes [4]. These fungi are also used as biological agents to control plant pathogens in agriculture [5, 6]. Since these organisms have a wide spectrum of applications, Embrapa (the Brazilian Agricultural Research Corporation) maintains an extensive collection of isolates of *Trichoderma* strains. The evaluation and selection of these strains in terms of their ability to produce cellulases is of considerable economic importance; however an efficient and reliable methodology is required for the screening of such a large number of isolates. 

Screening of cellulase-producing microorganisms can be performed on agar plates using a cellulosic substrate such as Avicel or carboxymethylcellulose (CMC) as carbon source for microorganism growth. Indicators used include dyes such as Congo red [7–9], Gram's iodine [10], Remazol Brilliant Blue R [11, 12], and mixtures of insoluble chromogenic substrates [13]. However, most of the studies using initial screening on plates have focused on qualitative evaluation of the potential of the strains to produce cellulolytic enzymes. There is, therefore, a need to correlate qualitative results from screening with quantitative results obtained for cellulase production during cultivation in a fermentation process. A microplate-based screening method to assess the cellulolytic potential of *Trichoderma* strains has recently been reported [14] and the results were compared with those obtained using submerged shake flask cultivations. Since the natural habitats of these filamentous fungi are solid media, the SSF procedure offers considerable advantages over submerged fermentation (SmF). These include high volumetric productivity, higher concentrations of the enzymes produced, and lower energy consumption [15, 16]. Another important feature of SSF is that it can use agroindustrial residues as carbon sources [15, 17]. To the best of our knowledge, at the present time there have been no studies reported concerning correlation between the results obtained by screening using plates and SSF cultivation.

Given the growing demand for the development of fast and simple procedures for prediction of the cellulase-producing potential of a given microorganism, this work aims to correlate qualitative results from the plate screening of filamentous fungi of the genus *Trichoderma*, with quantitative results obtained for cellulase production during cultivation under SSF. To evaluate the efficiency and reliability of this methodology for the screening of a large number of isolated microorganisms, 78 isolates of filamentous fungi from an extensive collection of isolates of *Trichoderma* strains maintained by Embrapa (the Brazilian Agricultural Research Corporation) were used. 

## 2. Materials and Methods

### 2.1. Trichoderma Isolates

A total of 78 strains of *Trichoderma* from Embrapa's mycology collection (Embrapa Meio Ambiente, Jaguariuna, Brazil, and Embrapa Recursos Geneticos, Brasilia, Brazil) were used in this study. For use as a reference strain, the cellulolytic mutant *T. reesei* Rut C30 was purchased from the Centre for Agricultural Bioscience International (CABI) culture collection in the UK (IMI number: 345108). All isolates were kept in potato dextrose agar (PDA) at 4°C and transferred to new PDA Petri plates at 30°C for short-term use.

### 2.2. Screening Step on Agar Plates Containing Avicel

The 78 strains were initially screened based on their ability to grow on a synthetic medium containing Avicel (Fluka Biochemika, Switzerland) as the sole carbon source. The composition of the medium was as follows: NaNO_3_ (3.0 g·L^−1^); K_2_HPO_4_ (1.0 g·L^−1^); MgSO_4_ (0.5 g·L^−1^); KCl (0.5 g·L^−1^); FeSO_4_·7H_2_O (0.01 mg·L^−1^); agar (20.0 g·L^−1^); Avicel (5.0 g·L^−1^). The pH was adjusted to 5.0 prior to sterilization. Using a sterile pipette tip, discs (diameter ~1 cm) were removed from plates containing the spores grown on PDA and placed onto the center of plates containing the Avicel medium. The experiment was performed using two replicates per strain.

### 2.3. Screening Step Using the Congo Red Test

The medium used for the second screening step using the Congo red test was similar to that described above (Section 2.2), except that the carbon source was low viscosity carboxymethylcellulose (CMC) (Sigma, USA). Only those strains that showed substantial growth in the initial screening with Avicel were selected for the Congo red test. Inoculation was carried out by using a platinum needle to transfer the spores from the PDA plate to the center of the plates containing the CMC medium [18]. The inoculated plates were incubated for 96 h at 30°C and the growth of the microorganism was measured by the diameter of the colony. A 10 mL aliquot of Congo red dye (2.5 g·L^−1^) was then added to each plate. After 15 min, the solution was discarded and the cultures were washed with 10 mL of 1 mol·L^−1^ NaCl. Cellulase production was indicated by the appearance of a pale halo with orange edges, indicative of areas of hydrolysis. This halo was measured for subsequent calculation of the enzymatic index (EI) using the expression:

(1) $$\begin{array}{c} \text{EI}=\frac{\text{diameter}\;\text{of}\;\text{hydrolysis}\;\text{zone}}{\text{diameter}\;\text{of}\;\text{colony}}. \end{array}$$

The strains that showed an EI higher than 1.50 were considered to be potential producers of cellulases. Three independent experiments were performed for this screening step, with two replicates per strain. For each strain the average EI of the three experiments was calculated, together with the standard deviation.

### 2.4. Screening Step Using Fermentation in Tubes

The composition of the liquid medium used for the fermentation in tubes was similar to that described in Section 2.2, except that the carbon source was a strip of Whatman No. 1 filter paper with dimensions 1 × 10 cm (corresponding to a mass of about 90 mg). Each tube was inoculated aseptically with a full loop of fungal spores from 9- to 10-day cultures grown on PDA plates [18]. Only those strains that had previously been selected using the Congo red test were evaluated in this step. Inoculated tubes were incubated for 4 weeks at 30°C, following the methodology described by Ruegger and Tauk-Tornisielo [18]. After this period, the filter paper containing the grown cells was slightly macerated in the liquid medium for desorption of the enzymes. Every week one tube of each strain was removed for enzymatic analysis.

### 2.5. Solid-State Fermentation (SSF)

Strains selected using the fermentation tube assay were cultivated under SSF. The SSF substrate was 5 g of a mixture of sugarcane bagasse and wheat bran at a ratio of 1 : 1. The initial moisture content of the substrate was adjusted to 80% using the nutrient medium [19]. Conidial suspensions of the *Trichoderma* strains were prepared by adding 20 mL of the nutrient medium to each plate and dislodging the spores into it by gentle pipetting. The spore count was adjusted to around 10^7^ spores per gram. All the SSF cultures were incubated at 30°C for 8 days in an incubator equipped with a forced air recirculation system (Tecnal, Brazil) and a container filled with water inside the chamber. The extraction was carried out by adding 100 mL of distilled water to each flask and agitating at 120 rpm for 40 min at 30°C. The mixture was then filtered and centrifuged. The clear supernatant was collected from each tube and assayed for enzyme activity.

### 2.6. Enzyme Assay

The enzymatic activity of EGase in the extracts was quantified and the results were expressed as activity units per gram of dry substrate. The cellulolytic activity of endoglucanase was determined by the CMC method [20]. Samples of the enzyme complexes (0.5 mL) were incubated together with 0.5 mL of the CMC substrate (4%) at 50°C for 10 min. After incubation, 1.0 mL of DNS was added to the tubes for quantification of the reducing sugars [21]. 

## 3. Results and Discussion

### 3.1. Initial Screening on Agar Plates Containing Avicel

Initial screening of the 78 *Trichoderma* strains was carried out by assessment of fungal growth on plates containing Avicel microcrystalline cellulose as the sole carbon source. A total of 49 test strains (62.8% of all strains evaluated), as well as the reference strain (*T. reesei* RUT C30), were able to hydrolyze this cellulosic substrate and exhibited obvious growth. The 29 strains that showed no potential for Avicel degradation were discarded at this stage.

Avicel has been used to measure exoglucanase activity; however the synergistic action of several different cellulases is required for the enzymatic hydrolysis of microcrystalline cellulose [2, 22]. Previous work using filamentous fungi employed selection based primarily on good growth of strains on Avicel and CMC. Of the 64 strains included in the screening, 25 were chosen as they presented good growth on these commercial substrates, with slightly better results obtained using CMC [23].

### 3.2. Screening Using the Congo Red Test

**Figure 1:**
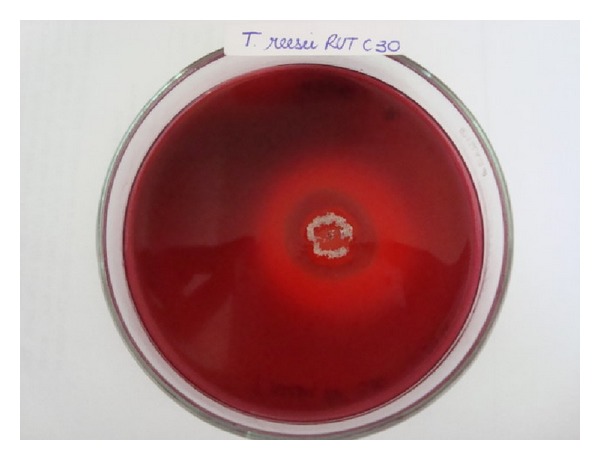
Observation of the clear zone around a colony of *T. reesei *Rut C30 using Congo red dye.

The second screening of the 49 previously selected strains was carried out using the Congo red test. Moreover two strains that showed no potential for Avicel degradation were tested for comparison of results. This test is based on the observation of growth and measurement of the hydrolysis halo that is used for calculation of the enzymatic index (EI). The halo produced by hydrolysis of cellulose is directly related to the region of action of cellulolytic enzymes, since the dye only remains attached to regions where there are *β*-1,4-D-glucanohydrolase bonds [24]. The pale halo around the colonies (Figure 1), which corresponds to the zone of CMC degradation, was observed for 48 strains (equivalent to 97.9% of the strains evaluated).

According to Ten et al. [13] the diameter of the halo zone is useful for selection of strains that can efficiently degrade polysaccharides such as cellulose, xylan, and amylose. Moreover, the enzymatic index can be used as a simple and rapid methodology to select strains within the same genus that have potential for the production of enzymes [18].  Table 1 shows the EI results obtained for cultivation of the fungi in synthetic medium containing CMC as sole carbon source, after 4 days of incubation at 30°C. The values given represent the average of measurements for 3 experiments performed independently under the same conditions. The strains that showed EI above 1.50 were as follows: *T. koningii* CEN 142 (EI = 1.90); *T. harzianum* CEN 139 (EI = 1.74); *T. *sp 104 NH (EI = 1.72); *T. *sp CEN 156 (EI = 1.64); *T. harzianum* CEN 241 (EI = 1.63); *T*. *harzianum* CEN 155 (EI = 1.61); *T. harzianum *CEN 238 (EI = 1.57); *T. *sp CEN 159 (EI = 1.56);  *T. koningii* CEN 201 (EI = 1.51); *T. harzianum* CEN 248 (EI = 1.51), *T.* sp CEN 90 (EI = 1.01), and *T*. sp CEN 97 (EI = 1.05). The EI value for the reference strain (*T. reesei* Rut C30) was 2.98.

The between-isolate variability of EI was low for those strains where an obvious hydrolysis halo was visible. The population mean was 1.41, with a standard deviation of 0.10. Ten strains (equivalent to 22.4% of the total number of strains) displayed EI values greater than 1.50 and were selected for the fermentation tube step.

Earlier work employed testing on plates using Congo red and calculation of enzymatic indices to determine the xylanolytic potential of eight strains of *Trichoderma* [25]. Strains T666 and T300 presented index values of 1.04 and 1.08, respectively. Although these values were not considered high, the authors noted good growth of the colonies of *Trichoderma* after 96 h. Ruegger and Tauk-Tornisielo [18] used enzymatic indices determined according to the Congo red procedure to evaluate the cellulolytic ability of 80 strains of fungi isolated from the soil of an ecological reserve in São Paulo, Brazil. Eighteen of the strains isolated belonged to the *Trichoderma* genus; however only four of these strains presented cellulolytic activity, with index values varying between 1.1 and 6.0. According to the authors, the EI value was suitable for the selection of strains belonging to the same genus. In other recent work [10], CMC Congo red plates presented low hydrolysis zone intensities when compared to a methodology using Gram's iodine reagent for selection of bacteria-producing cellulases. Nonetheless, allowance of a longer time for reaction of the dye with the medium could increase the visibility of hydrolysis zones, while the diameter of the halo can aid in selection of strains possessing high polysaccharide degradation activity [13].

### 3.3. Fermentation in Tubes

After screening of the cellulolytic potential of the fungi using Petri plates, ten strains were selected for quantitative evaluation using tube fermentations. This system simulates submerged fermentation with an insoluble substrate. If the organism remained in the liquid phase it would not have access to an element essential for its growth, namely, carbon [18]. If present in the solid phase, other nutrients can also be captured by diffusion through the medium (e.g., by absorption of liquid by filter paper). 

**Figure 2:**
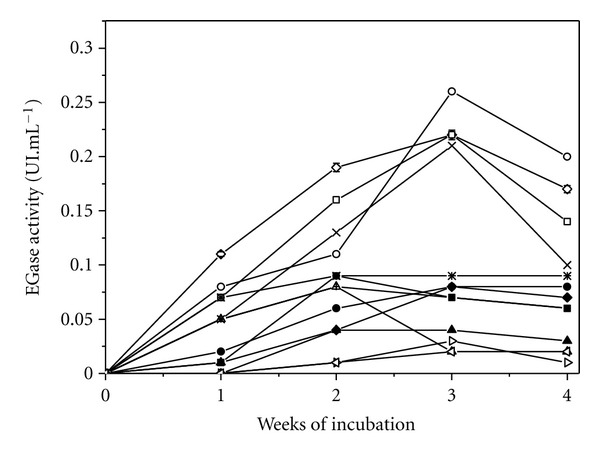
Production of EGase in tubes: (-○-) *T. harzianum *CEN 139; (-□-) *T. harzianum* CEN 155; (-*⋄*-) *T. *sp104 NH; (-×-) *T. reesei* Rut C30; (**-∗-**) *T. asperelum* CEN 201; (**-●-**) *T. harzianum* CEN 241; (-◆-) *T. harzianum *CEN 248; (-■-) *T. harzianum *CEN 238; (-+-) *T. *sp CEN 156; (-▲-) *T. koningii* CEN 142; (-∆-) *T. *sp CEN 159; (-*▹*-) *T. *sp CEN 90; (-*◃* -) *T. *sp CEN 97.

The tubes were incubated for four weeks and every week one tube was removed for analysis. EGase activities are shown in Figure 2 (as averages of two analyses). In contrast to experiments conducted using Petri plates, quantitative evaluation of cellulolytic potential can be achieved using tubes. It was observed that *T. harzianum* CEN 139, *T. *sp CG 104 NH, and *T. harzianum* CEN 155 showed the highest EGase activities, reaching 0.27, 0.23, and 0.22 UI·mL^−1^, respectively, after three weeks of fermentation. The strains that presented the highest enzymatic index values in the Congo red test (*T. harzianum* CEN 139 (EI = 1.74) and *T*. sp CG 104 NH (EI = 1.72)) also showed the highest endoglucanase activities. The hypercellulolytic reference strain (*T. reesei* Rut C30) showed an EGase activity of 0.22 UI·mL^−1^. Two of the test strains (CEN 139 and CG 104NH) showed greater enzymatic activities than that of the reference, while one strain (CEN 155) showed an EGase activity equal to that of the reference strain. As a negative control, the strains *T*. sp CEN 90 and CEN 97 were evaluated by tubes fermentation and presented the lowest values for the production of enzymes. These strains were also the ones with lowest EI.

According to Ruegger and Tauk-Tornisielo [18], *T. harzianum* V and *T. pseudokoningii* II presented values of 1.64 and 1.45 UI·mL^−1^, respectively, for endoglucanase activity in fermentation tubes. The values reported were higher than those obtained in the present study; however, it is important to note that the measured cellulase activity depends not only on the techniques employed, but also on the strains used. Although the genus may be the same (*Trichoderma*), the species may be different. 

**Figure 3:**
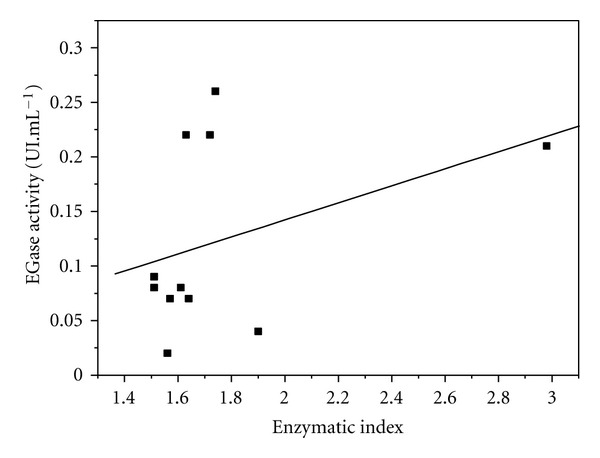
EGase production (UI·mL^−1^) versus enzymatic index of strains selected for fermentation in tubes.

Tube fermentation was used to quantitatively determine the cellulolytic potential of each individual strain. This type of fermentation test is especially interesting because it requires minimal quantities of nutrients. However, no direct correlation was obtained between the results of this procedure and the Congo red test (Figure 3).

### 3.4. Solid-State Fermentation

**Figure 4:**
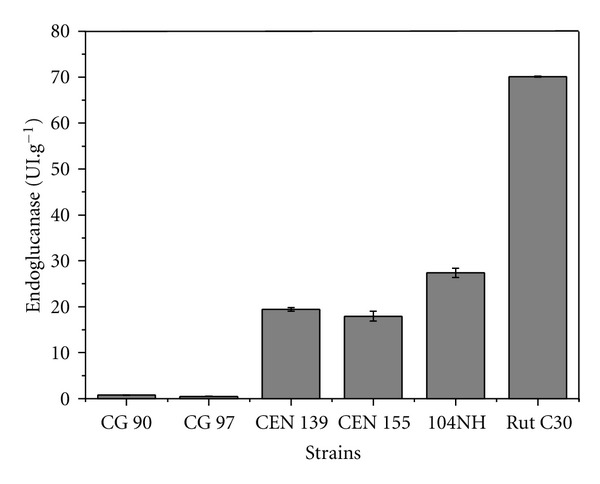
Production of EGase using solid-state fermentation of selected *Trichoderma* strains and comparison with *T. reesei* Rut C30.

The strains *T. harzianum* CEN 139, *T. harzianum* CEN 155, and *T*. sp CG 104NH were evaluated by cultivation under solid-state fermentation during a period of 8 days, using a substrate of sugarcane bagasse and wheat bran. This selected condition was based on preliminary experiments. These strains were selected because they showed the highest levels of endoglucanase production during fermentation in tubes. As mentioned before, the tube test system used simulates submerged fermentation with an insoluble carbon source. Since the majority of industrial process for enzyme production employs submerged fermentation, it was of interest to select strains that also showed a good performance under this cultivation system. The three strains showed similar endoglucanase activities, with the highest value (25.93 UI·g^−1^) obtained for *T*. sp CG 104NH. EGase production by the mutant strain (*T. reesei* Rut C30) was substantially higher than that of the other strains (70.24 UI·g^−1^) (Figure 4). As a negative control, the strains *T*. sp CEN 90 and CEN 97 were tested and similar to the other evaluations, the results were not significant (0.75 and 0.45 UI·g^−1^).

Several different substrates have been used previously for production of EGase by *Trichoderma* using SSF. Gutierrez-Correa and Tengerdy [26] measured endoglucanase production of 18.8 UI·g^−1^ by *Trichoderma reesei* LM-UC4 and 22.6 UI·g^−1^ by *Trichoderma reesei* LM-UC4E1 in SSF with sugarcane bagasse as substrate. In other recent work [27], EGase production of 25.2 UI·g^−1^ was obtained using *Trichoderma reesei* Rut C30 in SSF with a substrate of kinnow pulp and wheat bran. 

### 3.5. Correlation between the Congo Red Technique and Solid-State Fermentation

**Figure 5:**
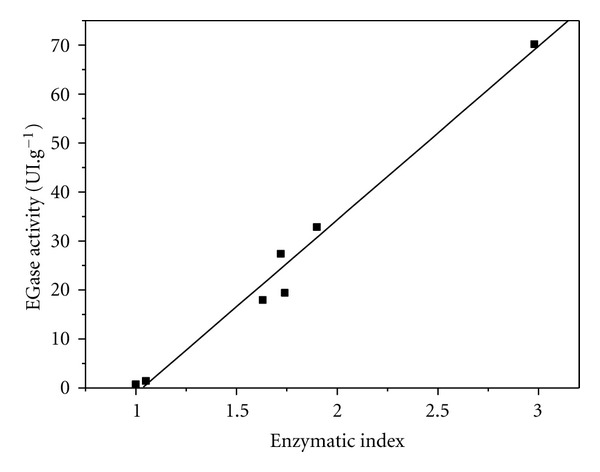
EGase production (UI·g^−1^) versus enzymatic index of strains selected for solid-state fermentation.

Species of the genus *Trichoderma* are recognized for their potential as biocontrol agents against phytopathogens [5, 28], as well as for their production of hydrolytic enzymes [29]. The establishment of a procedure for the selection of strains from a large population is therefore of great interest. The results obtained using screening on plates and SSF enzyme production were compared in order to identify any relationships between the techniques (Figure 5). A high correlation (*R* ^2^ = 0.977) was observed between the EI values obtained in the Congo red experiment and the endoglucanase activity values measured using solid-state fermentation. The EGase activities and enzymatic indices were in the ranges 17.9–32.8 UI·g^−1^ and 1.63–1.91, respectively, while the hypercellulolytic reference strain (*T. reesei* RUT C30) showed an EGase activity of 70.1 UI·g^−1^ and an enzymatic index of 2.98. The two strains (CEN 90 and 97) with lowest values to production of EGase were the strains with lowest enzymatic indices, 0.75–1.45 UI·g^−1^ and 1.0, respectively. As a positive control, SSF cultivations with the strain 142, which showed the highest enzymatic index among the strains from Embrapa's mycology collection (EI of 1.9), presented EGase activity of 32.86 ± 2.78 IU/g, thus contributing to consolidate our findings.

These results demonstrate the existence of a linear correlation between the two methodologies. Screening on plates employing Congo red dye, followed by a solid-state fermentation analysis, is, therefore, an effective method for selection of cellulase-producing strains of *Trichoderma*.

## 4. Conclusions

The qualitative assessment by screening in plates with different substrates showed to be feasible for both the ability of the strains hydrolyze microcrystalline cellulose medium, Avicel and amorphous cellulose, CMC through Congo red test. These methods in plates presented feasibility as they can be employed for an initial selection of strains, tests in addition to being simple, rapid and well adapted for screening of a large number of samples. The quantitative tests demonstrated a directed relationship with the qualitative, since the strains with higher EI showed higher production of EGase. The SSF was performed with the three strains largest producers of endoglucanase selected by the process on fermentation in tubes and showed significant results to production of EGase considering the substrate used in process (BC) and the strains are wild-type strains. It was possible to verify this study the correlation between the qualitative methodology in plates using Congo red and quantitative methodology by the process solid-state fermentation. The results obtained showed that the qualitative and quantitative methods are valid and important to selection strains of an extensive collection of the same species of microorganisms.

## Figures and Tables

**Table 1:** Enzymatic indices of filamentous fungi belonging to the genus *Trichoderma*.

Strain	Mean Øc*	Mean Øh**	EI	SD***
Rut C30	10.0	29.8	2.98	0.190
90	9.1	9.2	1.01	0.010
97	12.9	13.5	1.05	0.019
139	15.3	27.6	1.74	0.215
145	15.2	22.0	1.45	0.055
151	18.8	27.3	1.46	0.103
153	19.7	27.4	1.39	0.087
155	18.6	29.5	1.61	0.118
156	18.0	26.4	1.64	0.078
159	17.3	26.5	1.56	0.200
167	19.8	27.0	1.36	0.077
142	16.4	30.2	1.90	0.233
162	18.4	27.1	1.47	0.035
201	16.1	24.0	1.51	0.183
202	16.3	23.2	1.45	0.280
209	17.4	24.2	1.39	0.121
210	17.1	24.1	1.41	0.084
219	18.8	27.9	1.48	0.031
223	23.0	28.2	1.22	0.021
237	15.8	23.3	1.48	0.039
238	17.2	26.7	1.57	0.137
240	12.7	18.4	1.45	0.056
241	10.1	17.2	1.63	0.131
242	13.8	20.3	1.48	0.044
248	15.4	23.3	1.51	0.071
02	23.8	32.4	1.36	0.053
03	27.8	33.5	1.24	0.251
05	21.8	30.9	1.42	0.044
06	24.9	34.3	1.38	0.060
11	24.7	31.9	1.31	0.148
50	25.1	34.7	1.39	0.072
51	30.6	37.0	1.21	0.070
58	26.7	36.6	1.37	0.034
58′	26.2	36.7	1.40	0.082
67	25.2	29.2	1.16	0.048
71	25.4	32.8	1.29	0.036
73	27.5	33.9	1.25	0.111
87	21.1	*X*	0.00	*X*
88	22.4	31.1	1.40	0.245
92	24.3	35.2	1.45	0.224
94	18.9	25.5	1.36	0.127
98C	22.5	30.3	1.34	0.048
98D	24.1	32.3	1.35	0.089
100NH	23.3	26.4	1.13	0.026
104NH	16.2	27.9	1.72	0.197
111	21.1	27.9	1.32	0.086
124	29.5	33.5	1.27	0.040
128	25.5	31.4	1.24	0.116
140	22.5	26.9	1.20	0.061
141	23.6	32.6	1.39	0.072
141′	26.0	32.8	1.28	0.133
144	28.1	36.1	1.29	0.077

*Øc: halo colonies; **Øh: halo hydrolysis; ***SD: standard deviation.
